# Additive effect of admission hyperglycemia on left ventricular stiffness in patients following acute myocardial infarction verified by CMR tissue tracking

**DOI:** 10.1186/s12933-024-02295-y

**Published:** 2024-06-20

**Authors:** Pei-Lun Han, Kang Li, Yu Jiang, Yue Gao, Ying-Kun Guo, Zhi-Gang Yang, Yuan Li

**Affiliations:** 1 Department of Radiology and West China Biomedical Big Data Center, West China Hospital, Sichuan University, Chengdu, China; 2 Med-X Center for Informatics, Sichuan University, Chengdu, China; 3 Shanghai Artificial Intelligence Laboratory, Shanghai, China; 4 Department of Radiology, West China Second University Hospital, Sichuan University, Chengdu, China

**Keywords:** Stress hyperglycemia, Acute myocardial infarction, Left ventricle, Strain, Magnetic resonance imaging

## Abstract

**Background:**

Stress hyperglycemia occurs frequently in patients following acute myocardial infarction (AMI) and may aggravate myocardial stiffness, but relevant evidence is still lacking. Accordingly, this study aimed to examine the impact of admission stress hyperglycemia on left ventricular (LV) myocardial deformation in patients following AMI.

**Methods:**

A total of 171 patients with first AMI (96 with normoglycemia and 75 with hyperglycemia) underwent cardiac magnetic resonance (CMR) examination were included. AMI patients were classified according to admission blood glucose level (aBGL): < 7.8 mmol/L (*n* = 96), 7.8–11.1 mmol/L (*n* = 41) and ≥ 11.1 mmol/L (*n* = 34). LV strains, including global radial/circumferential/longitudinal peak strain (PS)/peak systolic strain rate (PSSR)/peak diastolic strain rate (PDSR), were measured and compared between groups. Further, subgroup analyses were separately conducted for AMI patients with and without diabetes. Multivariate analysis was employed to assess the independent association between aBGL and LV global PS in AMI patients.

**Results:**

LV global PS, PSSR and PDSR were decreased in radial, circumferential and longitudinal directions in hyperglycemic AMI patients compared with normoglycemic AMI patients (all *P* < 0.05). These differences were more obvious in patients with diabetes than those without diabetes. AMI patients with aBGL between 7.8 and 11.1 mmol/L demonstrated significant decreased radial and longitudinal PS, radial PSSR, and radial and longitudinal PDSR than those with aBGL < 7.8 mmol/L (all *P* < 0.05). AMI patients with aBGL ≥ 11.1 mmol/L showed significantly decreased PS, PSSR and PDSR in all three directions than those with aBGL < 7.8 mmol/L, and decreased longitudinal PSSR than those with aBGL between 7.8 and 11.1 (all *P* < 0.05). Further, aBGL was significantly and independently associated with radial (β = − 0.166, *P* = 0.003) and longitudinal (β = 0.143, *P* = 0.008) PS.

**Conclusions:**

Hyperglycemia may exacerbate LV myocardial stiffness in patients experienced first AMI, leading to reduction in LV strains. aBGL was an independent indicator of impaired LV global PS in AMI patients. Blood glucose monitoring is more valuable for AMI patients with diabetes.

**Supplementary Information:**

The online version contains supplementary material available at 10.1186/s12933-024-02295-y.

## Background

Stress hyperglycemia is a common occurrence among patients admitted for acute myocardial infarction (AMI) regardless of their diabetes status [1, 2]. Admission blood glucose level (aBGL) often serves as a valuable metric reflecting the underlying glucometabolic dysfunction and quantifying the presence of stress hyperglycemia [3, 4]. There is a growing acknowledgment that elevated aBGL is linked to a heightened risk of adverse outcomes within the context of AMI [5, 6]. The additive effect of stress hyperglycemia on left ventricular (LV) function may provide potential explanations for this negative association [7, 8]. Myocardial stiffness, as an inherent property of the myocardium, represents the resistance of the myocardium to deformation and affects both diastolic and systolic cardiac function [9–11]. It is well known that both AMI and hyperglycemia status can increase LV myocardial stiffness, and thus damage LV function [12, 13]. A better understanding of the interactions between hyperglycemia and cardiac change in AMI patients is of great importance to facilitate patient management and improve outcomes.

Cardiac magnetic resonance (CMR) is considered the gold standard for evaluating cardiac morphology, function, and myocardial tissue, owing to its exceptional soft-tissue contrast and temporal resolution [14, 15]. In recent years, the utilization of CMR tissue tracking has enabled the measurement of myocardial deformation through tracking of myocardial motion, reflecting myocardial stiffness and exhibiting a high level of sensitivity in detecting subclinical myocardial dysfunction [16]. However, to the best of our knowledge, the impact of admission hyperglycemia on CMR-derived LV strains in patients with AMI remains unexplored [17]. Therefore, the objective of this study was to verify whether admission stress hyperglycemia aggravates the LV myocardial deformation in AMI patients by CMR tissue tracking, and to investigate the association between aBGL and LV global strain.

## Methods

### Study population

This study retrospectively screened 703 consecutive patients who were admitted for AMI and underwent CMR examinations between January 2012 and January 2023. The diagnosis of AMI was made based on the European Society of Cardiology (ESC)/American College of Cardiology (ACC)/American Heart Association (AHA)/World Heart Federation (WHF) expert consensus criteria [18]. All AMI patients received treatment according to current clinical recommendations [19, 20], and CMR was performed before discharge. The exclusion criteria encompassed the following: (1) previous myocardial infarction (a clinical diagnosis of old MI, a previous discharge diagnosis of MI, or previous symptoms of MI) or coronary revascularization (either percutaneous coronary intervention or coronary artery bypass graft surgery); (2) coexisting acquired cardiomyopathy, severe valvular disease necessitating surgical intervention, or congenital heart disease; (3) severe renal failure (estimated glomerular filtration rate < 30 mL/min); and (4) poor image quality. Finally, a total of 171 patients with first AMI (137 males and 34 females; mean age 56.71 ± 12.46 years) were included in the study. Then, according to the presence of admission stress hyperglycemia (aBGL of ≥ 7.8 mmol/L) [3], the AMI patients were classified as normoglycemia (*n* = 96) and hyperglycemia (*n* = 75) groups.

### Clinical data

Basic information, including demographic information, clinical characteristics, serum biochemical indexes and treatment, were retrospectively collected from the medical records. The serum biochemical indexes were evaluated within a week of the CMR examination except for the aBGL and the peak troponin level.

### CMR scanning protocol

All subjects underwent CMR scans on 3.0-T whole-body magnetic resonance imaging scanners (Tim Trio/ MAGNETOM Skyra; Siemens Medical Solutions, Erlangen, Germany) in a supine position. A dedicated two-element cardiac-phased array coil was attached, and a standard ECG-triggering device was concurrently employed. A serious of 8–12 continuous short-axis view of cine images, and two-chamber, three-chamber and four-chamber long-axis views of cine images were obtained using a balanced steady-state free-precession sequence (repetition time (TR): 2.8/ 3.4 ms; echo time (TE): 1.22/ 1.20 ms; flip angle: 40°/ 50°; slice thickness: 8 mm; field of view (FOV): 250 × 300/ 340 × 285 mm^2^; matrix size: 208 × 139/ 256 × 166 pixels). Gadolinium-based contrast agent (0.2 mL/kg, 2.5–3.0 mL/s) was injected intravenously followed by saline flush (20 mL, 3.0 mL/s). After 10–15 min, a segmented-turbo-FLASH–phase-sensitive inversion recovery sequence (TR: 750/ 512 ms; TE: 1.18/ 1.24 ms; flip angle: 20°/ 40°; slice thickness: 8 mm; FOV: 240 × 300/ 288 × 360 mm^2^; matrix size: 256 × 184/ 256 × 125 pixels) was used for LGE imaging.

### Imaging analysis

The CMR data were imported into a dedicated software (CVI42, Circle Cardiovascular Imaging, Inc., Calgary, Canada) for imaging analyses. The CMR measurements were conducted by two experienced radiologists blinded to the patients’ clinical information separately. In case of discordance between their findings, they engaged in a discussion and arrived at a consensus.

### LV structure and function analysis

We manually delineated the endocardial and epicardial contours of the left ventricle in serial short-axis slices at the end-diastolic and end-systolic phases, and then the LV end-diastolic volume (LVEDV), LV end-systolic volume (LVESV), LV stroke volume (LVSV), LV ejection fraction (LVEF) and LV mass were automatically obtained. The LV mass was indexed to body surface area to obtain the LV mass index (LVMI). The LV remodeling index (LVRI) was determined as the ratio of LV mass to LVEDV.

**Fig. 1:**
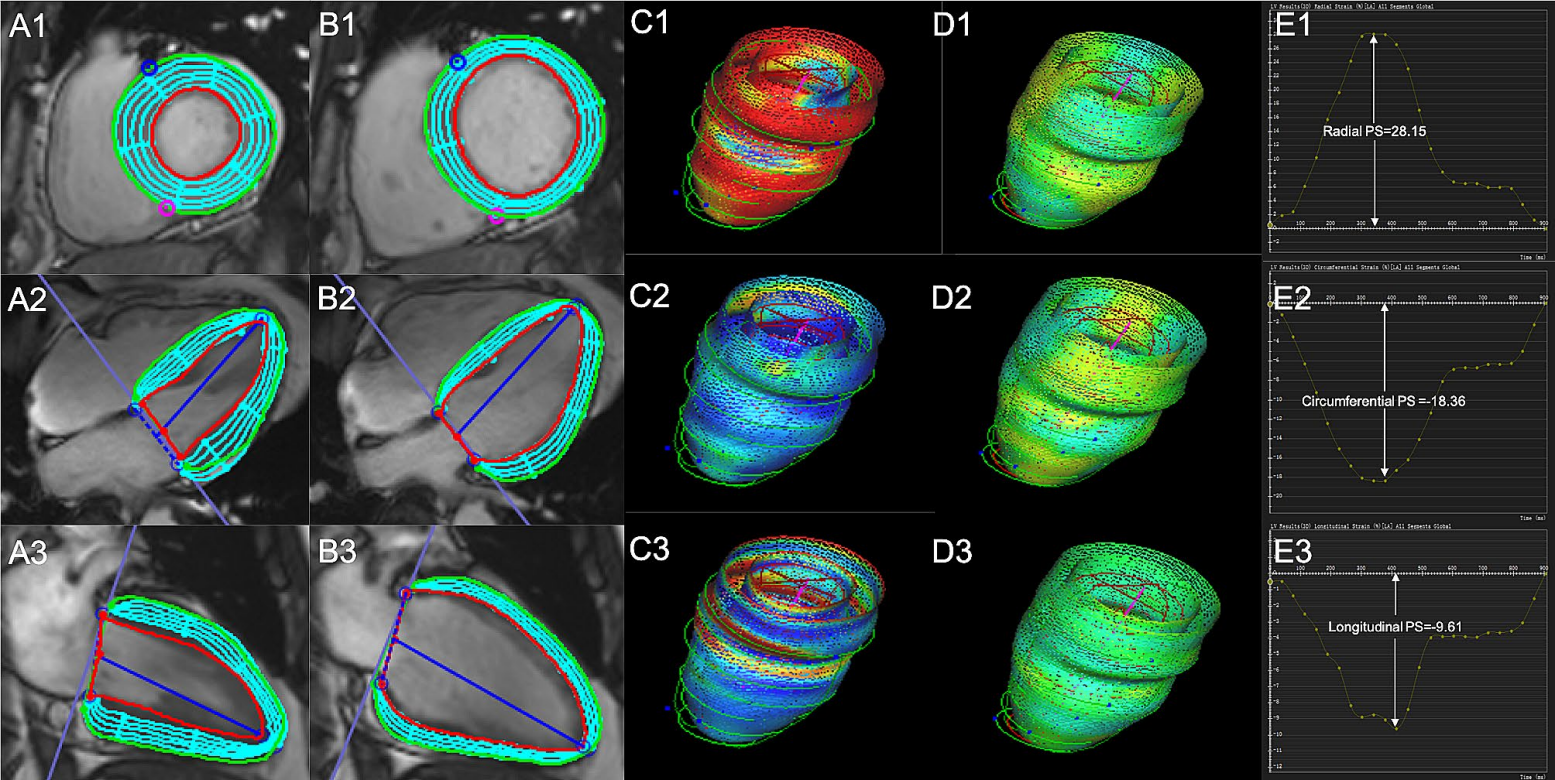
CMR tissue tracking derived LV strain analysis. **A1–A3, B1–B3** Epi- and endocardial left ventricle borders were traced in long-axis two-chamber, four-chamber, and serial short-axis slices at the end-systolic and end-diastolic phases; **C1–C3, D1–D3** Three-dimensional pseudo-color images of the LV global radial, circumferential, and longitudinal PS at the end-systolic and end-diastolic phases; **E1–E3** CMR-derived LV global PS curves in radial, circumferential, and longitudinal directions. *CMR* Cardiac magnetic resonance, *LV *Left ventricular, *PS *Peak strain

The CMR tissue tracking technique was used for the analysis of LV myocardial strain. The endocardial and epicardial contours of the left ventricle were manually delineated in long-axis two-chamber, long-axis four-chamber, and serial short-axis slices during the end-systolic and end-diastolic phases. Then, the three-dimensional tissue tracking model was automatically generated using the end-diastole as the reference phase (Fig. 1). The LV global peak strain (PS), peak systolic strain rate (PSSR) and peak diastolic strain rate (PDSR) in the radial, circumferential, and longitudinal directions were automatically derived.

### LGE images analysis

The myocardial scar was identified as a signal intensity that exceeded the mean of normal myocardium by five standard deviations [21]. By contouring the endocardial and epicardial borders of the left ventricle on the LGE short-axis images and using normal-appearing myocardium as a reference, the global myocardium infarct mass was obtained. Infarct size was expressed as the percentage of infarct mass to LV mass. The LV infarct territory was analyzed by dividing it into the interventricular septum, anterior wall, inferior wall, and lateral wall based on the AHA 16-segment model [22]. Microvascular obstruction (MVO) was defined as a dark zone within the LGE regions [23].

### Intra- and interobserver reproducibility analysis

After 1 month, LV global PS was once again measured in 20 randomly selected cases by the same radiologist to evaluate the intraobserver variability. In addition, a second blinded radiologist measured LV global PS to determine the interobserver variability.

### Statistical analysis

All statistical analyses were conducted with SPSS 23.0 (SPSS, Inc.) and GraphPad Prism 10 (GraphPad Prism, Inc.). The normality of the data was evaluated using the Kolmogorov–Smirnov test. Continuous variables were presented as the mean ± standard deviation (normally distributed) or the median with interquartile range (IQR) (nonnormally distributed). Categorical variables were presented as frequencies (%). For continuous variables, one-way analysis of variance followed by LSD post hoc test or Kruskal-Wallis test followed by Mann-Whitney U test was performed to compare three groups, and Student’s t test or Mann-Whitney U test was used to compare two groups as appropriate. Categorical variables were compared using Chi-square tests or Mann-Whitney U tests (ordered categorical data). Correlations between aBGL and LV global PS were analyzed by Pearson or Spearman correlation analysis. Univariable linear regression analyses were performed to demonstrate the relationship between candidate factors and LV global PS. Age, sex and variables with a P value < 0.1 in the univariable analyses were entered into a stepwise multivariable linear regression analysis. The stepwise multiple linear regression can avoid multicollinearity among variables, which is its inherent issue [24, 25]. The inter- and intra-observer variabilities for reproducibility were evaluated using the intraclass correlation coefficient (ICC). For all statistical analyses, a P value < 0.05 was considered statistically significant.

## Results

### Baseline characteristics

Demographic information, clinical characteristics, serum biochemical indices and treatment findings are reported in Table 1. The distribution of age and sex was comparable among the normoglycemic and hyperglycemic groups. Notably, known diabetes [31 (41.3%) vs. 11 (11.5%), *P* < 0.001], insulin usage [7 (9.3%) vs. 0 (0.0%), *P* = 0.008], and biguanides usage [7 (9.3%) vs. 1 (1.0%), *P* = 0.029] were more frequent in AMI patients with hyperglycemia than in those without hyperglycemia. The AMI with hyperglycemia group showed markedly higher aBGL [10.45 (8.40, 14.44) vs. 6.47 (5.66, 6.92), *P* < 0.001] and glycosylated hemoglobin A1c levels [6.40 (5.80, 8.55) vs. 5.85 (5.50, 6.25), *P* = 0.003] than the normoglycemic group. In addition, the number of obstructive diseased vessels in the hyperglycemic group was significantly higher than that in the normoglycemic group [1.00 (1.00, 2.00) vs. 1.00 (0.00, 1.00), *P* = 0.044].

**Table 1:** Baseline characteristics of the study cohort

	Normoglycemia (*n* = 96)	Hyperglycemia (*n* = 75)	*P*
Age, years	55.70 ± 13.10	58.00 ± 11.56	0.232
Sex, n (%)			0.674
Female	18 (18.8%)	16 (21.3%)	
Male	78 (81.3%)	59 (78.7%)	
BMI, kg/m^2^	24.58 (21.11, 27.27)	25.95 (22.86, 29.39)	0.089
SBP, mmHg	125.43 ± 22.14	128.93 ± 23.56	0.319
DBP, mmHg	77.02 ± 13.46	80.27 ± 16.10	0.153
HR, bpm	75.10 (67.28, 83.98)	75.40 (68.20, 82.70)	0.338
Cardiovascular risk factors, n (%)			
Smoking	59 (61.5%)	48 (64.0%)	0.733
Drinking	43 (44.8%)	35 (46.7%)	0.807
Hyperlipidemia	12 (12.5%)	6 (8.0%)	0.341
Hypertension	47 (49.0%)	38 (50.7%)	0.825
Diabetes	11 (11.5%)	31 (41.3%)	< 0.001
Family History	16 (16.7%)	7 (9.3%)	0.163
Killip functional class, n (%)			0.822
I	69 (71.9%)	54 (72.0%)	
II	19 (19.8%)	10 (13.3%)	
III	6 (6.3%)	9 (12.0%)	
IV	2 (2.1%)	2 (2.7%)	
Laboratory results			
Peak troponin, ng/L	2207.00 (735.50, 5991.00)	3325.00 (1030.00, 9036.00)	0.235
aBGL, mmol/L	6.47 (5.66, 6.92)	10.45 (8.40, 14.44)	< 0.001
HbA1C, mmol/L (nmiss = 86)	5.85 (5.50, 6.25)	6.40 (5.80, 8.55)	0.003
Total cholesterol, mmol/L	4.25 ± 0.97	4.51 ± 1.11	0.745
Triglycerides, mmol/L	1.31 (0.93, 1.82)	1.52 (0.99, 2.46)	0.086
HDL, mmol/L	1.15 ± 0.29	1.18 ± 0.34	0.851
LDL, mmol/L	2.42 (2.00, 3.06)	2.50 (2.01, 3.08)	0.563
eGFR, mL/min/1.73m^2^	76.00 (65.25, 87.70)	76.00 (67.00, 89.00)	0.905
AMI subtype, n (%)			0.174
STEMI	61 (63.5%)	55 (73.3%)	
NSTEMI	35 (36.5%)	20 (26.7%)	
Lesion location			
LM	7 (7.3%)	8 (10.7%)	0.439
LAD	77 (80.2%)	64 (85.3%)	0.382
LCx	44 (45.8%)	40 (53.3%)	0.330
RCA	52 (54.2%)	50 (66.7%)	0.098
No. of diseased vessels	2.00 (1.00, 3.00)	2.00 (2.00, 3.00)	0.079
No. of obstructive vessels	1.00 (0.00, 1.00)	1.00 (1.00, 2.00) †	0.044
No. of non-obstructive vessels	1.00 (0.00, 2.00)	1.00 (0.00, 1.00)	0.851
Concomitant medication, n (%)			
ACEI/ARB	4 (4.2%)	6 (8.0%)	0.464
β-blockers	5 (5.2%)	3 (4.0%)	0.995
Calcium-channel blocker	19 (19.8%)	12 (16.0%)	0.523
Diuretics	1 (1.0%)	3 (4.0%)	0.447
Aspirin	1 (1.0%)	0 (0%)	1.000
Statin	2 (2.1%)	0 (0%)	0.505
Insulin	0 (0%)	7 (9.3%)	0.008
Biguanides	1 (1.0%)	7 (9.3%)	0.029
α-Glucosidase inhibitor	1 (1.0%)	4 (5.3%)	0.232
PCI treatment, n (%)	57 (59.4%)	54 (72.0%)	0.086
Period from AMI onset to CMR, days	4.00 (2.00, 6.00)	3.00 (2.00, 5.00)	0.222

*BMI b*ody mass index, *SBP* systolic blood pressure, *DBP* diastolic blood pressure, *HR* heart rate, *aBGL* Admission blood glucose level, *HbA1C* Glycosylated hemoglobin A1c, *LDL* low-density lipoprotein, *eGFR* estimated glomerular filtration rate, *AMI* acute myocardial infarction, *STEMI* ST-segment elevation myocardial infarction, *NSTEMI* non-ST-segment elevation myocardial infarction, *LM* left main, *LAD* left anterior artery, *LCx l*eft circumflex, *RCA* Right coronary artery, *ACEI* Asngiotensin converting enzyme inhibitor, *ARB* Angiotensin receptor blockers, *PCI* percutaneous coronary intervention, *CMR* cardiac magnetic resonance

### Comparison of CMR indicates between AMI patients with and without hyperglycemia

**Fig. 2:**
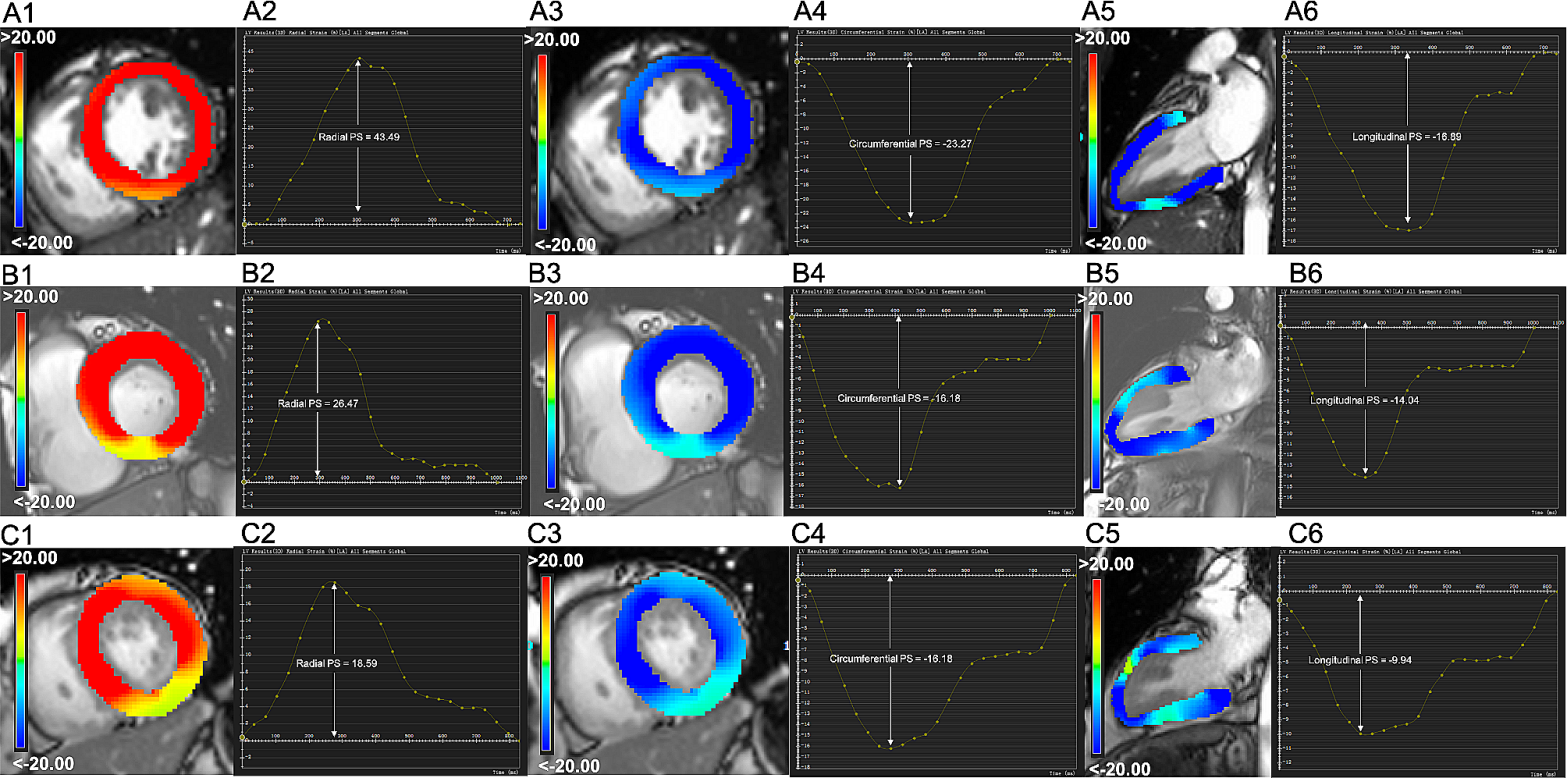
Representative CMR pseudocolor images at end-systole and CMR‐derived LV global PS curves in radial, circumferential, and longitudinal directions derived from a normal control (**A1–A6**), a normoglycemic AMI patient (**B1**–**B6**), and a hyperglycemic AMI patient (**C1–C6**). *AMI* Acute myocardial infarction, *CMR* Cardiac magnetic resonance, *LV* Left ventricular, *PS* Peak strain

There were no significant differences in LVEF and other traditional LV structure and function indicators such as LVEDV, LVESV, LVSV, LVMI, and LVRI between the hyperglycemic and normoglycemic cases (all *P* > 0.05) (Table 2). Regarding LV strains, LV global PS, PSSR, and PDSR in the radial, circumferential and longitudinal directions were significantly decreased in hyperglycemic AMI patients compared with normoglycemic AMI patients (all *P* < 0.05) (Fig. 2). Additionally, hyperglycemic AMI patients had significantly increased infarct size (30.64 ± 15.43 vs. 24.84 ± 15.73, *P* = 0.018) and more MVO lesions [36 (48.0%) vs. 31 (32.3%), *P* = 0.037] than normoglycemic patients.

**Table 2:** Comparison of CMR findings between controls, normoglycemic AMI patients, and hyperglycemic AMI patients

	Normoglycemia (*n* = 96)	Hyperglycemia (*n* = 75)	*P*
LVEDV, mL	150.55 (127.45, 165.38)	152.60 (121.95, 182.05)	0.418
LVESV, mL	72.14 (51.73, 104.21)	77.12 (59.04, 103.29)	0.463
LVSV, mL	70.82 ± 21.22	71.11 ± 22.99	0.934
LVEF, %	48.01 ± 14.10	46.29 ± 14.30	0.431
LVMI, g/m^2^	56.48 (45.94, 67.87)	54.81 (49.12, 71.01)	0.746
LVRI, g/mL	0.73 ± 0.19	0.73 ± 0.20	0.988
Peak strain, %			
Radial	29.06 ± 12.68	22.02 ± 11.87	< 0.001
Circumferential	− 13.07 ± 4.28	− 11.32 ± 3.97	0.006
Longitudinal	−10.95 ± 3.71	−8.86 ± 3.45	< 0.001
PSSR, 1/s			
Radial	1.73 (1.27, 2.47)	1.19 (0.84, 1.78)	< 0.001
Circumferential	− 0.89 ± 0.32	− 0.77 ± 0.26	0.008
Longitudinal	− 0.72 (− 0.93, − 0.57)	− 0.59 (− 0.81, − 0.44)	0.001
PDSR, 1/s			
Radial	− 1.94 (− 2.90, − 1.26)	− 1.20 (− 1.97, − 0.79)	< 0.001
Circumferential	0.80 ± 0.29	0.69 ± 0.26	0.014
Longitudinal	0.66 (0.51, 0.83)	0.55 (0.39, 0.68)	0.001
Infarct size, % of LV	24.84 ± 15.73	30.64 ± 15.43	0.018
Infarct territory, n (%)			
Anterior	29 (30.2%)	25 (33.3%)	0.663
Inferior	37 (38.5%)	29 (38.7%)	0.987
Interventricular septum	60 (62.5%)	51 (68.0%)	0.455
Lateral	21 (21.9%)	22 (29.3%)	0.265
Transmural infarction, n (%)	25 (26.0%)	27 (36.0%)	0.160
MVO, n (%)	31 (32.3%)	36 (48.0%)	0.037

*CMR* cardiac magnetic resonance, *AMI a*cute myocardial infarction, *LV* left ventricular, *EF* ejection fraction, *EDV* end diastolic volume, *ESV* end systolic volume, *SV* stroke volume, *EF* ejection fraction, *MI m*ass index, *RI* remodeling index, *PS* peak strain, *PSSR* peak systolic strain rate, *PDSR* peak diastolic strain rate, *MVO* microvascular obstruction

### Effect of hyperglycemia on CMR indicators in AMI patients with and without diabetes

Then, patients were stratified by diabetes status and divided into two subgroups: non-diabetes (*n* = 129) and diabetes (*n* = 42). Comparisons of clinical characteristics in non-diabetic and diabetic patients with and without hyperglycemia are reported in Additional file 1: Table S1. Further, among AMI patients without diabetes, hyperglycemic patients exhibited a numerically decrease in LV radial, circumferential and longitudinal PS, circumferential and longitudinal PSSR, and circumferential and longitudinal PDSR (all *P* > 0.05) and a notable decrease in LV radial PSSR and PDSR (both *P* < 0.05) (Table 3). These differences were more obvious in diabetic subgroup.

**Table 3:** CMR findings of diabetic and non-diabetic AMI patients, according to hyperglycemia

	Non-diabetes (*n* = 129)		*P*	Diabetes (*n* = 42)		*P*
	Normoglycemia (*n* = 85)	Hyperglycemia (*n* = 44)		Normoglycemia (*n* = 11)	Hyperglycemia (*n* = 31)	
LVEDV, mL	148.95 (127.51, 166.28)	147.62 (118.40, 175.57)	0.823	152.89 ± 30.77	170.62 ± 51.63	0.292
LVESV, mL	71.91 (53.12, 101.13)	69.62 (54.55, 85.64)	0.481	83.32 ± 39.06	104.82 ± 53.59	0.231
LVSV, mL	70.76 ± 21.84	73.51 ± 23.74	0.512	71.33 ± 16.45	67.70 ± 21.82	0.619
LVEF, %	50.83 (38.10, 57.29)	49.15 (40.60, 60.09)	0.728	49.29 ± 16.72	40.88 ± 14.74	0.124
LVMI, g/m^2^	56.16 (45.25, 67.73)	52.60 (46.80, 61.53)	0.374	62.06 ± 17.50	64.16 ± 15.03	0.705
LVRI, g/mL	0.72 ± 0.18	0.71 ± 0.17	0.721	0.75 ± 0.22	0.75 ± 0.24	0.976
Peak strain, %						
Radial	28.68 ± 12.21	24.85 ± 12.90	0.100	31.97 ± 16.26	18.00 ± 8.98	0.001
Circumferential	− 13.12 ± 4.06	− 12.46 ± 3.77	0.371	− 12.70 ± 5.95	− 9.69 ± 3.45	0.048
Longitudinal	− 10.88 ± 3.65	− 9.57 ± 3.44	0.052	− 11.56 ± 4.33	− 7.84 ± 3.26	0.005
PSSR, 1/s						
Radial	1.72 (1.27, 2.36)	1.45 (0.94, 1.94)	0.023	2.45 (1.30, 3.22)	0.96 (0.75, 1.61)	0.001
Circumferential	−0.89 ± 0.32	− 0.85 ± 0.24	0.502	− 0.94 ± 0.38	− 0.65 ± 0.25	0.008
Longitudinal	−0.77 ± 0.28	−0.72 ± 0.28	0.304	− 0.80 ± 0.39	− 0.53 ± 0.21	0.006
PDSR, 1/s						
Radial	− 1.78 (− 2.90, − 1.26)	− 1.53 (− 2.11, − 0.93)	0.013	− 2.58 (− 3.01, -1.00)	− 0.91 (− 1.68, − 0.75)	0.010
Circumferential	0.79 ± 0.29	0.77 ± 0.26	0.694	0.90 ± 0.28	0.59 ± 0.23	0.001
Longitudinal	0.66 (0.50, 0.81)	0.56 (0.44, 0.73)	0.060	0.75 ± 0.22	0.53 ± 0.20	0.004
Infarct size, % of LV	24.65 ± 15.25	30.96 ± 16.04	0.031	26.31 ± 19.85	30.18 ± 14.77	0.500
Infarct territory, n (%)						
Anterior	26 (30.6%)	13 (29.5%)	0.903	3 (27.3%)	12 (38.7%)	0.754
Inferior	32 (37.6%)	15 (34.1%)	0.691	5 (45.5%)	14 (45.2%)	1.000
Interventricular septum	53 (62.4%)	27 (61.4%)	0.913	7 (63.6%)	24 (77.4%)	0.621
Lateral	17 (20.0%)	8 (18.2%)	0.804	4 (36.4%)	14 (45.2%)	0.879
Transmural infarction, n (%)	23 (27.1%)	12 (27.3%)	0.979	2 (18.2%)	15 (48.4%)	0.163
MVO, n (%)	28 (32.9%)	21 (47.7%)	0.101	3 (27.3%)	15 (48.4%)	0.389

*CMR* cardiac magnetic resonance, *AMI* acute myocardial infarction, *LV* left ventricular, *EF e*jection fraction, *EDV* end diastolic volume, *ESV* end systolic volume, *SV* stroke volume, *EF* ejection fraction, *MI m*ass index, *RI* Remodeling index, *PS* Peak strain, *PSSR* peak systolic strain rate, *PDS*R peak diastolic strain rate, *MVO* Microvascular obstruction

### CMR characteristics of AMI patients with different aBGL levels

AMI patients were classified into three groups based on a statement by American Heart Association [3] to investigate whether LV strain and strain rate decrease with the increase of aBGL. Among AMI patients, 96 patients (56.1%) had aBGL < 7.8 mmol/L, 41 patients (24.0%) had aBGL between 7.8 and 11.1 mmol/L and 34 patients (19.9%) had aBGL ≥ 11.1 mmol/L (Table 4).

**Table 4:** CMR findings of AMI patients with different admission blood glucose level

	Blood glucose level on admission (mmol/l)			*P*
	< 7.8 (*n* = 96)	7.8 − 11.1 (*n* = 41)	≥ 11.1 (*n* = 34)	
LVEDV, mL	150.55 (127.45, 165.38)	151.07 (121.3, 176.87)	155.41 (124.22, 204.39)	0.635
LVESV, mL	72.14 (51.73, 104.21)	70.26 (56.18, 89.54)	85.71 (61.35, 127.40)	0.389
LVSV, mL	70.82 ± 21.22	74.62 ± 22.26	66.87 ± 23.48	0.314
LVEF, %	48.01 ± 14.10	49.40 ± 13.41	42.54 ± 14.64	0.082
LVMI, g/m^2^	56.48 (45.94, 67.87)	54.12 (47.67, 69.94)	58.27 (50.33, 74.37)	0.604
LVRI, g/mL	0.73 ± 0.19	0.72 ± 0.17	0.74 ± 0.24	0.912
Peak strain, %				
Radial	29.06 ± 12.68	24.15 ± 12.20 *	19.45 ± 11.11*	< 0.001
Circumferential	− 13.07 ± 4.28	− 11.83 ± 3.81	− 10.69 ± 3.90*	0.011
Longitudinal	− 10.95 ± 3.71	− 9.20 ± 3.42 *	− 8.45 ± 3.50*	0.001
PSSR, 1/s				
Radial	1.73 (1.27, 2.47)	1.33 (0.95, 1.94) *	1.00 (0.78, 1.64) *	< 0.001
Circumferential	− 0.89 ± 0.32	− 0.80 ± 0.22	− 0.73 ± 0.30 *	0.018
Longitudinal	−0.72 (− 0.93, − 0.57)	− 0.67 (− 0.90, − 0.50)	− 0.58 (− 0.65, − 0.39) *†	< 0.001
PDSR, 1/s				
Radial	−1.94 (− 2.90, − 1.26)	−1.61 (− 2.21, − 0.90) *	− 0.95 (− 1.74, − 0.76) *	< 0.001
Circumferential	0.80 ± 0.29	0.74 ± 0.26	0.64 ± 0.25 *	0.017
Longitudinal	0.66 (0.51, 0.83)	0.56 (0.44, 0.73) *	0.53 (0.39, 0.64) *	< 0.001
Infarct size, % of LV	24.84 ± 15.73	31.01 ± 16.20	30.19 ± 14.67	0.057
Infarct territory, n (%)				
Anterior	29 (30.2%)	11 (26.8%)	14 (41.2%)	0.375
Inferior	37 (38.5%)	16 (39.0%)	13 (38.2%)	0.997
Interventricular septum	60 (62.5%)	21 (51.2%) *	30 (88.2%) *	0.003
Lateral	21 (21.9%)	11 (26.8%)	11 (32.4%)	0.462
Transmural infarction, n (%)	25 (26.0%)	12 (29.3%)	15 (44.1%)	0.142
MVO, n (%)	31 (32.3%)	21 (51.2%)	15 (44.1%)	0.093

*CMR* Cardiac magnetic resonance, *AMI* Acute myocardial infarction, *LV* Left ventricular, *EF *Ejection fraction, *EDV* End diastolic volume, *ESV* End systolic volume, *SV* Stroke volume, *EF* Ejection fraction, *MI* Mass index, *RI* Remodeling index,* PS* Peak strain, *PSSR* peak systolic strain rate, *PDSR *peak diastolic strain rate, *MVO* Microvascular obstruction

* *P* < 0.05 versus patients with blood glucose level < 7.8 mmol/L; † *P* < 0.05 versus patients with blood glucose level between 7.8 and 11.1 mmol/L

In patients with 7.8 ≤ aBGL < 11.1 mmol/L, the PS (radial and longitudinal), PSSR (radial) and PDSR (radial and longitudinal) were lower than those with aBGL < 7.8 mmol/L (all *P* < 0.05). In patients with aBGL ≥ 11.1 mmol/L, the PS, PSSR and PDSR in all three directions were decreased compared to those with aBGL < 7.8 mmol/L (all *P* < 0.05). When comparing the aBGL ≥ 11.1 mmol/L and 7.8 ≤ aBGL < 11.1 mmol/L groups, the longitudinal PSSR was further significantly decreased (*P* = 0.046), and other LV stain indices were slightly decreased but not statistically significant (all *P* > 0.05). In addition, there was a significant difference in the presence of interventricular septal infarction among these three groups [for patients with aBGL < 7.8 vs. 7.8–11 vs. ≥ 11.1 mmol/l: 60 (62.5%) vs. 21 (51.2%) vs. 30 (88.2%), *P* = 0.003]. No appreciable difference was found in other CMR indicators among these groups.

### Factors related to LV global PS in AMI patients

**Fig. 3:**
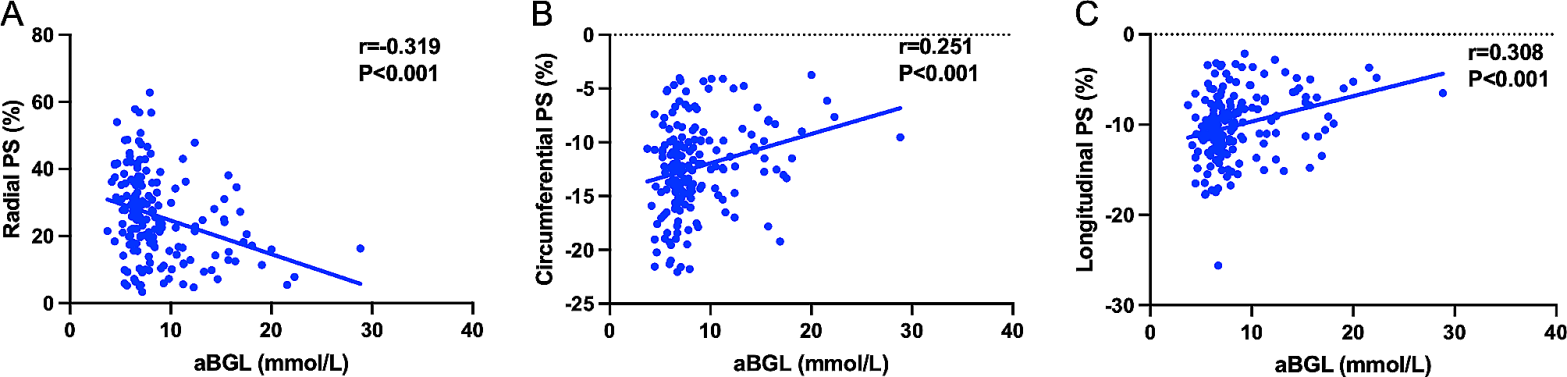
Correlations between aBGL and the LV global radial **A**, circumferential **B**, and longitudinal **C** PS in AMI patients. *aBGL* admission blood glucose level, *LV* left ventricular, *PS* Peak strain, *AMI* Acute myocardial infarction

Regarding association between aBGL and LV global PS in AMI patients, spearman correlation analysis showed that there was a significant correlation between aBGL and LV global radial PS (*r* = − 0.319, *P* < 0.001), circumferential PS (*r* = 0.251, *P* < 0.001) and longitudinal PS (*r* = 0.308, *P* < 0.001) in patients with AMI (Fig. 3).The univariate linear regression analysis in Table 5 demonstrated that aBGL was significantly associated with LV global PS in the radial (β = − 0.309, *P* < 0.001), circumferential (β = 0.257, *P* = 0.001) and longitudinal (β = 0.297, *P* < 0.001) directions. After adjusting for confounding factors, multivariate linear regression analysis further showed that aBGL was an independent risk factor for LV global radial (β = − 0.166, *P* = 0.003) and longitudinal (β = 0.143, *P* = 0.008) PS.

**Table 5:** Univariate and multivariate linear regression of LV global PS in all patients with AMI

	Radial PS				Circumferential PS				Longitudinal PS			
	Univariable		Multivariable (*R*^2^ = 0.512)		Univariable		Multivariable (*R*^2^ = 0.643)		Univariable		Multivariable (*R*^2^ = 0.551)	
	β	*P*	β	*P*	β	*P*	β	*P*	β	*P*	β	*P*
Age	0.006	0.943			0.067	0.386			0.049	0.525		
Sex	− 0.099	0.198			0.063	0.412			0.055	0.471		
BMI	0.032	0.677			0.032	0.678			0.065	0.397		
SBP	− 0.014	0.851			− 0.070	0.363			0.007	0.923		
DBP	− 0.168	0.028	−0.148	0.007	0.082	0.287			0.087	0.257		
HR	− 0.232	0.002			0.242	0.001			0.215	0.005		
Diabetes	− 0.193	0.011			0.250	0.001	0.131	0.006	0.187	0.014		
aBGL	− 0.309	< 0.001	− 0.166	0.003	0.257	0.001			0.297	< 0.001	0.143	0.008
eGFR	0.004	0.960			− 0.055	0.476			− 0.056	0.468		
No. of obstructive vessels	− 0.256	0.001			0.269	< 0.001			0.303	< 0.001		
Insulin	− 0.128	0.096			0.099	0.198			0.150	0.050		
Biguanides	− 0.085	0.268			0.104	0.175			0.087	0.258		
LVEF	0.686	< 0.001	0.686	< 0.001	− 0.779	< 0.001	− 0.709	< 0.001	− 0.724	< 0.001	− 0.641	< 0.001
Infarct size	− 0.355	< 0.001			0.458	< 0.001	0.189	< 0.001	0.422	< 0.001	0.123	0.003
Anterior infarction	− 0.185	0.016			0.170	0.026			0.225	0.003		
Interventricular septum infarction	− 0.153	0.046			0.167	0.029	− 0.118	0.019	0.253	0.001		
MVO	− 0.314	< 0.001			0.381	< 0.001			0.356	< 0.001		

*PS* peak strain, *AMI* acute myocardial infarction, *BMI* Body mass index, *SBP* systolic blood pressure, *DBP* diastolic blood pressure, *HR *heart rate, *HbA1C* glycosylated hemoglobin A1c, *aBGL* admission blood glucose level, *MVO* microvascular obstruction

Besides, LVEF (β = 0.686, *P* < 0.001) and DBP (β = − 0.148, *P* = 0.007) were significantly and independently associated with LV radial PS. Diabetes (β = 0.131, *P* = 0.006), LVEF (β = − 0.709, *P* < 0.001), infarct size (β = 0.189, *P* < 0.001), and interventricular septum infarction (β = − 0.118, *P* = 0.019) were independent factors associated with LV circumferential PS. LVEF (β = − 0.641, *P* < 0.001) and infarct size (β = 0.123, *P* = 0.003) were significantly and independently associated with LV longitudinal PS.

### Reproducibility of LV global PS

The CMR tissue tracking technique for measuring LV global PS had robust inter- and intraobserver agreements (ICC (95% CI) = 0.996 (0.990–0.998) and 0.993 (0.983–0.997), respectively, for LV radial PS; ICC (95% CI) = 0.998 (0.995–0.999) and 0.998 (0.994–0.999), respectively, for LV circumferential PS; ICC (95% CI) = 0.994 (0.985–0.998) and 0.986 (0.966–0.995), respectively, for LV longitudinal PS).

## Discussion

Our study focused on the impact of admission stress hyperglycemia on LV myocardial stiffness in patients following a first AMI. The principal findings were as follows: (1) Compared with normoglycemic AMI patients, hyperglycemic patients, especially those with diabetes, presented more severe damage with respect to LV strains in the radial, circumferential and longitudinal directions, despite comparable LVEF; (2) Along with the increase in aBGL, the LV strain values of the AMI patients decreased progressively; (3) aBGL was an independent indicator of LV global radial and longitudinal PS in AMI patients; and (4) Diabetes, infarct size, and interventricular septum infarction were independent factors associated with LV circumferential PS in AMI patients. In addition, infarct size was also an independent factor associated with LV longitudinal PS.

Admission stress hyperglycemia is a frequently occurring transient metabolic condition and represents an important risk factor for the development of adverse cardiovascular events [1, 4, 26]. In patients with AMI, admission hyperglycemia is common (20–50% depending on the definition of hyperglycemia), and a heightened risk of adverse cardiovascular outcomes has been reported among AMI patients accompanied by admission hyperglycemia [1, 27, 28]. The cardiac myocardial edema and inflammation or fibrosis triggered following AMI leads to adverse cardiac remodeling and LV wall motion abnormalities [29, 30]. Meanwhile, stress hyperglycemia has also been reported to be associated with poor cardiac wall motion [12]. Ishihara et al.’s [7] study revealed a notable decrease in LVEF among AMI patients with hyperglycemia compared to those without hyperglycemia. Consistently, Paolisso et al. [28] corroborated this finding in their study.

In our results, LVEF did not differ between normoglycemic and hyperglycemic AMI patients. This difference might be due to the different characteristics of the included populations or the different time intervals from the onset of AMI to related auxiliary examinations. As mentioned above, there have been some studies [7, 28] on the relationship between hyperglycemia at admission and cardiac function, however, they mainly focused on traditional indicators, such as LVEF, derived from echocardiography or angiography. Nevertheless, the present study employed a more sophisticated approach–CMR tissue tracking technique–for identifying LV dysfunction [31, 32] and demonstrated that LV global strains were significantly decreased in AMI patients with hyperglycemia compared with normoglycemic patients, even in the absence of significant LVEF difference. These findings suggest that the detrimental impact of hyperglycemia on LV stiffness in patients following AMI may occur prior to the decline in LVEF, thereby potentially enabling the early identification of patients at risk of developing cardiac dysfunction.

In the present study, the AMI patients with hyperglycemia had markedly decreased PS, PSSR and PDSR in the radial, circumferential and longitudinal directions compared to the corresponding values in AMI patients without hyperglycemia. A previous study [12] on hyperglycemic diabetes has demonstrated that stress hyperglycemia is a powerful predictor correlated with the reduction in LV contractile function. These findings indicate that concomitant admission stress hyperglycemia may have deleterious effects on myocardial stiffness in the context of AMI, rendering it more vulnerable to LV dysfunction. The heightened polyol pathway-mediated oxidative stress induced by hyperglycemia has been reported as the primary pathological mechanism of myocardial dysfunction [17, 33], which may partial explain the deterioration of LV stiffness in AMI. Tight glycemic control might reduce myocardial stiffness and improve cardiac outcome in AMI patients [34]. Further, the LV strain reduction was more worsening in patients with diabetes, and diabetes was an independent factor associated with impaired LV circumferential PS. Diabetes may promote myocardial fibrosis, LV stiffness, and cardiac dysfunction [30]. It might remind us to pay attention to the treatment for hyperglycemic AMI patients with diabetes.

By comparing AMI patients with different glucose levels on admission, our study revealed a progressive slightly decline in LV global PS, PSSR and PDSR in all three directions upon elevation of aBGL. Furthermore, multivariate analysis revealed that aBGL was an independent risk factor for LV global PS in the radial (β = − 0.166) and longitudinal (β = 0.143) directions, even after adjusting for diabetes. These findings suggest that LV global PS may decrease with increasing glucose levels on admission, and greater priority should be given to patients with aBGL ≥ 11.1 mmol/L, as myocardial stiffness was more pronounced in this population than that in those with lower aBGL. Additionally, it was observed that the correlation between LV global PS and aBGL exhibited greater strength in the radial direction. Based on previous published data [35], we speculated that the initial alteration in myocardial strain subsequent to hyperglycemia may involve a decrease in radial strain, which needs further confirmation in future studies.

Additionally, compared with that in normoglycemic patients, the LV strain-related damage primarily occurred along the radial and longitudinal directions in patients with 7.8 ≤ aBGL < 11.1 mmol/L. The lo ngitudinal PSSR was significantly decreased in patients with aBGL ≥ 11.1 mmol/L compared with patients with aBGL < 11.1 mmol/L. There are several reasons that could account for this phenomenon. First, the potential initial detrimental effects of hyperglycemia may manifest in radial strains [35]. Second, it is widely recognized that hyperglycemia impairs the endocardium, where the myocardium that produces longitudinal stress mainly exists [36]. Moreover, the circumferential strains began to significantly decline in patients with aBGL ≥ 11.1 mmol/L compared with normoglycemic patients, which may contribute to the poorer outcomes of AMI patients with higher aBGL [37].

Besides, the study showed that interventricular septum infarction was significantly associated with LV global radial, circumferential and longitudinal PS impairment. Further, interventricular septum infarction was an independent factor associated with the impairment of LV circumferential PS. The interventricular septum has the highest density of blood vessels within the heart, with left anterior artery suppling with 2/3 blood and posterior descending artery (80% originated from right coronary artery) suppling the remaining 1/3 [38]. When the culprit vessel involves these arteries, impairment of the blood supply may affect LV deformation. Infarct size had an independent association with LV circumferential and longitudinal PS. Circumferential myofibers are located within the midwall; therefore, a larger infarct size is more likely to cause the dysfunction in circumferential directions. Furthermore, longitudinal myofibers are more susceptible to myocardial ischemia due to epicardial artery stenosis, as they distributed within the subendocardium.

Our study had several limitations. First, it was a single-center study, and possible selection bias cannot be excluded. Multicenter studies are warranted to verify our results. Second, our study cannot establish causality between hyperglycemia and LV stiffness due to the nature of its cross-sectional design. Third, since glycosylated hemoglobin A1c was only tested in some but not all patients, the stress hyperglycemia ratio or the ratio of acute to chronic glycemic values etc [39]. cannot be calculated for all patients. Further studies are needed to evaluated the associations between these indicators and LV stiffness. Besides, we did not track with changes over time in the BGL. Further studies are expected to evaluate the impact of glycemic variability up on LV stiffness and function. Last, further studies are warrened to explore the association between admission hyperglycemia and follow-up cardiac strain and function.

## Conclusions

Hyperglycemia may aggravate LV myocardial stiffness in AMI patients, especially in those with known diabetes, leading to a reduction in LV strains, which could be detected prior to LVEF decline. Further, there was a significant independent association between elevated aBGL and decreased LV global PS, emphasizing the importance of glucose monitoring and management upon admission for patients following AMI. For AMI patients with diabetes, glucose monitoring is more valuable.

### Electronic supplementary material

Supplementary Material 1.

## Data Availability

No datasets were generated or analysed during the current study.

## Abbreviations

AMI: Acute myocardial infarction
aBGL: Admission blood glucose level
CMR: Cardiac magnetic resonance
ICC: Intraclass correlation coefficient
LV: Left ventricular
LVEDV: Left ventricular end-diastolic volume
LVESV: Left ventricular end-systolic volume
LVSV: Left ventricular stroke volume
LVEF: Left ventricular ejection fraction
LVMI: Left ventricular mass index
LVRI: Left ventricular remodeling index
LGE: Late gadolinium enhancement
MVO: Microvascular obstruction
PS: Peak strain
PSSR: Peak systolic strain rate
PDSR: Peak diastolic strain rate
