# Supplementary material for: Additive effect of admission hyperglycemia on left ventricular stiffness in patients following acute myocardial infarction verified by CMR tissue tracking
Source: Cardiovasc Diabetol. 2024 Jun 20;23:210. doi: 10.1186/s12933-024-02295-y (PMC11191232; doi:10.1186/s12933-024-02295-y)
Supplement: Supplementary file 1 — Supplementary Material 1. [file 12933_2024_2295_MOESM1_ESM.docx]

**Online-Only Additional file**

**Additive effect of admission hyperglycemia on left ventricular stiffness in patients following acute myocardial infarction verified by CMR tissue tracking**

**Table S1 Baseline characteristics of diabetic and non-diabetic AMI patients, according to hyperglycemia**

|  | Non-diabetes (n=129) | | P | Diabetes (n=42) | | P |
| --- | --- | --- | --- | --- | --- | --- |
|  | Normoglycemia (n=85) | Hyperglycemia (n=44) |  | Normoglycemia (n=11) | Hyperglycemia (n=31) |  |
| Age, years | 55.91 ± 13.15 | 56.27 ± 12.08 | 0.878 | 54.09 ± 13.16 | 60.45 ± 10.47 | 0.113 |
| Sex, n (%) |  |  | 0.804 |  |  | 0.463 |
| Female | 17 (20.0%) | 8 (18.2%) |  | 1 (9.1%) | 8 (25.8%) |  |
| Male | 68 (80.0%) | 36 (81.8%) |  | 10 (90.9%) | 23 (74.2%) |  |
| BMI, kg/m^2^ | 24.49 (21.23, 27.35) | 26.67 (23.92, 29.92) | 0.073 | 24.26 ± 5.14 | 25.33 ± 4.81 | 0.538 |
| SBP, mmHg | 125.25 ± 21.00 | 125.73 ± 22.86 | 0.905 | 126.82 ± 30.83 | 133.48 ± 24.15 | 0.469 |
| DBP, mmHg | 77.65 ± 13.81 | 77.84 ± 16.53 | 0.944 | 72.18 ± 9.53 | 83.71 ± 15.05 | 0.023 |
| HR, bpm | 75.00 (67.45, 86.25) | 71.85 (65.20, 78.53) | 0.143 | 75.80 (66.60, 78.30) | 79.00 (71.10, 85.30) | 0.233 |
| Cardiovascular risk factors, n (%) |  |  |  |  |  |  |
| Previous/current smoker | 51 (60.0%) | 27 (61.4%) | 0.881 | 8 (72.7%) | 21 (67.7%) | 1.000 |
| Drink | 37 (43.5%) | 19 (43.2%) | 0.970 | 6 (54.5%) | 16 (51.6%) | 0.867 |
| Hyperlipidemia | 9 (10.6%) | 1 (2.3%) | 0.184 | 3 (27.3%) | 5 (16.1%) | 0.718 |
| Hypertension | 39 (45.9%) | 20 (45.5%) | 0.963 | 8 (72.7%) | 18 (58.1%) | 0.618 |
| Family History | 12 (14.1%) | 2 (4.5%) | 0.174 | 4 (36.4%) | 5 (16.1%) | 0.328 |
| Killip functional class, n (%) |  |  | 0.682 |  |  | 0.365 |
| I | 64 (75.3%) | 32 (72.7%) |  | 5 (45.5%) | 22 (71.0%) |  |
| II | 14 (16.5%) | 6 (13.6%) |  | 5 (45.5%) | 4 (12.9%) |  |
| III | 5 (5.9%) | 6 (13.6%) |  | 1 (9.1%) | 3 (9.7%) |  |
| IV | 2 (2.4%) | 0 (0.0%) |  | 0 (0.0%) | 2 (6.5%) |  |
| Laboratory results |  |  |  |  |  |  |
| Peak troponin, ng/L | 3050.50 (869.28, 6645.50) | 3602.00 (1443.25, 9948.50) | 0.204 | 836.800 (398.00, 1750.00) | 2344.00 (630.20, 5395.00) | 0.041 |
| aBGL, mmol/L | 6.37 (5.64, 6.92) | 8.80 (8.15, 9.99) | <0.002 | 6.56 ± 0.68 | 15.05 ± 4.31 | <0.001 |
| HbA1C, mmol/L (nmiss = 86) | 5.80 (5.50, 6.10) | 5.80 (5.60, 5.98) | 0.746 | 6.82 ± 1.53 | 8.3 ± 1.61 | 0.069 |
| Total cholesterol, mmol/L | 4.30 ± 0.91 | 4.44 ± 1.03 | 0.445 | 3.82 ± 1.31 | 4.62 ± 1.22 | 0.073 |
| Triglycerides, mmol/L | 1.31 (0.91, 1.80) | 1.53 (0.92, 2.17) | 0.440 | 1.40 (1.05, 1.93) | 1.43 (1.25, 2.72) | 0.399 |
| HDL, mmol/L | 1.16 ± 0.28 | 1.20 ± 0.36 | 0.154 | 1.06 ± 0.35 | 1.16 ± 0.32 | 0.399 |
| LDL, mmol/L | 2.42 (2.00, 3.06) | 2.50 (2.01, 3.08) | 0.769 | 2.20 ± 1.02 | 2.63 ± 0.90 | 0.190 |
| eGFR, mL/min/1.73m^2^ | 77.00 (65.50, 87.70) | 74.50 (63.00, 83.50) | 0.328 | 75.00 (63.00, 87.00) | 78.00 (68.00, 98.00) | 0.463 |
| AMI subtype, n (%) |  |  | 0.143 |  |  | 0.676 |
| STEMI | 55 (64.7%) | 34 (77.3%) |  | 6 (54.4%) | 21 (67.7%) |  |
| NSTEMI | 30 (35.3%) | 10 (22.7%) |  | 5 (45.5%) | 10 (32.3%) |  |
| Lesion location |  |  |  |  |  |  |
| LM | 6 (7.1%) | 3 (6.8%) | 1.000 | 1 (9.1%) | 5 (16.1%) | 0.943 |
| LAD | 69 (81.2%) | 37 (84.1%) | 0.682 | 8 (72.7%) | 27 (87.1%) | 0.530 |
| LCx | 40 (47.1%) | 24 (54.5%) | 0.420 | 4 (36.4%) | 16 (51.6%) | 0.384 |
| RCA | 44 (51.8%) | 31 (70.5%) | 0.041 | 8 (72.7%) | 19 (61.3%) | 0.754 |
| No. of diseased vessels | 2.00 (1.00, 3.00) | 2.00 (2.00, 3.00) | 0.136 | 2.00 (1.00, 3.00) | 2.00 (1.00, 3.00) | 0.535 |
| No. of obstructive vessels | 1.00 (0.00, 1.00) | 1.00 (1.00, 2.00) | 0.085 | 1.00 (0.00, 2.00) | 1.00 (0.00, 2.00) | 0.693 |
| No. of non-obstructive vessels | 1.00 (0.00, 2.00) | 1.00 (0.00, 1.00) | 0.673 | 0.00 (0.00, 2.00) | 1.00 (0.00, 1.00) | 0.822 |
| PCI, n (%) | 50 (58.8%) | 34 (77.3%) | 0.037 | 7 (63.6%) | 20 (64.5%) | 1.000 |
| Concomitant medication, n (%) |  |  |  |  |  |  |
| ACEI/ARB | 4 (4.7%) | 3 (6.8%) | 0.927 | 0 (0.0%) | 3 (9.7%) | 0.554 |
| β-blockers | 5 (5.9%) | 1 (2.3%) | 0.630 | 0 (0.0%) | 2 (6.5%) | 1.000 |
| Calcium-channel blocker | 18 (21.2%) | 5 (11.4%) | 0.167 | 1 (9.1%) | 7 (22.6%) | 0.595 |
| Diuretics | 1 (1.2%) | 2 (4.5%) | 0.557 | 0 (0.0%) | 1 (3.2%) | 1.000 |
| Aspirin | 1 (1.2%) | 0 (0%) | 1.000 | 0 (0%) | 0 (0%) | NA |
| Statin | 2 (2.4%) | 0 (0%) | 0.547 | 0 (0%) | 0 (0%) | NA |
| Insulin | 0 (0%) | 0 (0%) | NA | 0 (0%) | 7 (22.6%) | 0.209 |
| Biguanides | 0 (0%) | 0 (0%) | NA | 1 (9.1%) | 7 (22.6%) | 0.595 |
| α-Glucosidase inhibitor | 0 (0%) | 0 (0%) | NA | 1 (9.1%) | 4 (12.9%) | 1.000 |
| Period from AMI onset to CMR, days | 4.00 (2.00, 6.50) | 3.00 (2.00, 6.00) | 0.264 | 5.00 (2.00, 6.00) | 3.00 (2.00, 7.00) | 0.516 |

AMI acute myocardial infarction, DM diabetes; BMI Body mass index, SBP systolic blood pressure, DBP diastolic blood pressure, HR heart rate, aBGL Admission blood glucose level, HbA1C Glycosylated hemoglobin A1c, LDL low-density lipoprotein, eGFR Estimated glomerular filtration rate, STEMI ST-segment elevation myocardial infarction, NSTEMI non-ST-segment elevation myocardial infarction, LM Left main, LAD left anterior artery, LCx Left circumflex, RCA Right coronary artery, PCI percutaneous coronary intervention, ACEI Asngiotensin converting enzyme inhibitor, ARB Angiotensin receptor blockers
